# Supplementary material for: High-fidelity DNA ligation enforces accurate Okazaki fragment maturation during DNA replication
Source: Nat Commun. 2021 Jan 20;12:482. doi: 10.1038/s41467-020-20800-1 (PMC7817679; doi:10.1038/s41467-020-20800-1)
Supplement: Supplementary file 1 — Supplementary Information [file 41467_2020_20800_MOESM1_ESM.pdf]

Supplementary Information

High-fidelity DNA ligation enforces accurate Okazaki fragment maturation during DNA replication

Jessica S. Williams, Percy P. Tumbale, Mercedes E. Arana, Julian A. Rana, R. Scott Williams and Thomas A. Kunkel

Correspondence: williamsrs@niehs.nih.gov or kunkel@niehs.nih.gov

PDF Contents:

Supplementary Fig. 1. Growth analysis of the *cdc9-EE/AA* mutant +/- MMR or *RAD27*

Supplementary Fig. 2. *URA3* mutation spectrum for the *cdc9-EE/AA msh2*Δ strain

Supplementary Fig. 3. *URA3* mutation spectrum for the *cdc9-EE/AA msh6*Δ strain

Supplementary Fig. 4. *URA3* mutation spectrum for the *cdc9-EE/AA msh3*Δ strain

Supplementary Fig. 5. *URA3* mutation spectrum for the *cdc9-EE/AA rad27*Δ strain

Supplementary Fig. 6. Cdc9 catalytic activity on control DNA substrates

Supplementary Fig. 7. Suppression of hLIG ligation fidelity on insC substrates

Supplementary Table 1. Haploid *S. cerevisiae* strains

Supplementary Table 2. Mutation events in the *cdc9-EE/AA* strain involving multiple bases

Supplementary Table 3. Total and specific mutation counts for the *cdc9-EE/AA* mutant strain ± MMR or *RAD27*

Supplementary Table 4. Mutation Specificity in the *MSH6* or *MSH3*-deficient strains +/- *cdc9-EE/AA*

Supplementary Table 5. Duplications observed in the *cdc9-EE/AA rad27*Δ strain

Supplementary Table 6. Breakdown of mutation classes in the *cdc9-EE/AA*, *cdc9-EE/AA rad27*Δ and *rad27*Δ strains

Supplementary Table 7. Duplications observed in the *rad27*Δ strain

Supplementary Table 8. Hotspot analysis of +1 G/C insertions in the low-fidelity *cdc9-EE/AA* mutant strain ± *MSH2*

Supplementary Table 9. Data collection and refinement statistics

Supplementary Table 10. Insertion and Control DNA oligonucleotides

Supplementary Table 11. Crystallization oligonucleotides

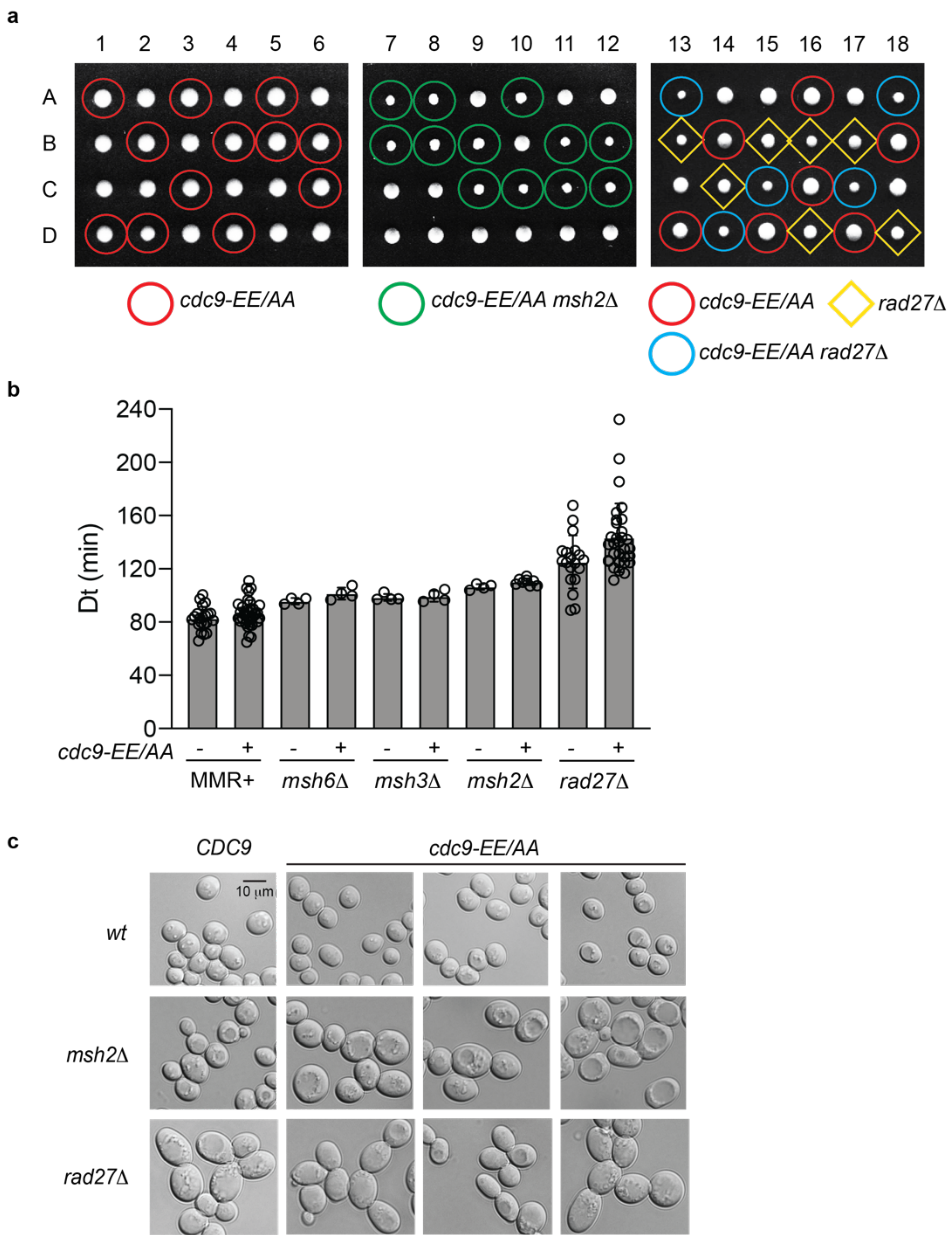

Supplementary Fig. 1. Growth analysis of the *cdc9-EE/AA* mutant +/- MMR or *RAD27*. a, Tetrad analysis of the *cdc9-EE/AA* (panel 1), *cdc9-EE/AA msh2*Δ (panel 2) and *cdc9-EE/AA rad27*Δ (panel 3) heterozygous diploids. Plates were photographed after 3 d growth at 30°C. **b**, Growth rate analysis of the *cdc9-EE/AA* mutant +/- *MSH6*, *MSH3*, *MSH2* or *RAD27* was performed by measuring the doubling times of cultures grown in rich medium at 30°C. Mean values are displayed (Biological replicates: n=21 for wt; n=35 for *cdc9-EE/AA*; n=4 for *msh6*Δ; n=4 for *cdc9-EE/AA msh6*Δ; n=4 for *msh3*Δ; n=4 for *cdc9-EE/AA msh3*Δ; n=4 for *msh2*Δ; n=8 for *cdc9-EE/AA msh2*Δ; n=19 for *rad27*Δ; n=31 for *cdc9-EE/AAA rad27*Δ). Error bars represent standard deviation. Source data is provided as a Source Data file. **c**, Representative microscopic images of live cells were captured following growth of the indicated strains to mid-log phase in YPDA at 30°C. This experiment was performed 3 times independently with similar results.

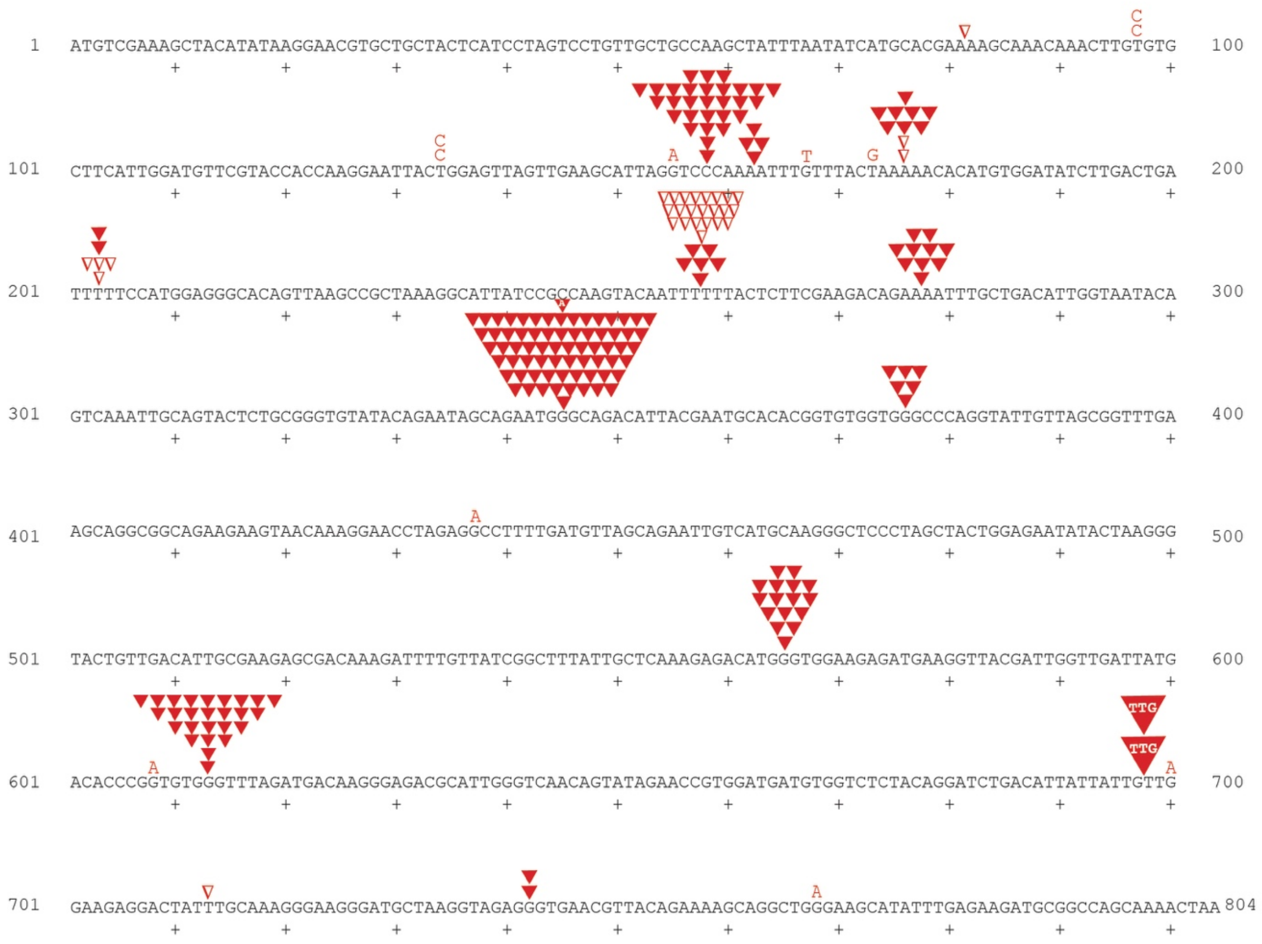

Supplementary Fig. 2. *URA3* mutation spectrum for the *cdc9-EE/AA msh2*Δ strain. The coding strand of the 804-bp *URA3* gene is shown. Sequence changes of 5 bp in size or less that are observed in independent *ura3* mutants are depicted in red. Letters indicate single-base substitutions, closed triangles indicate single-base additions, open triangles indicate single-base deletions. Large closed triangles indicate 3 base additions.

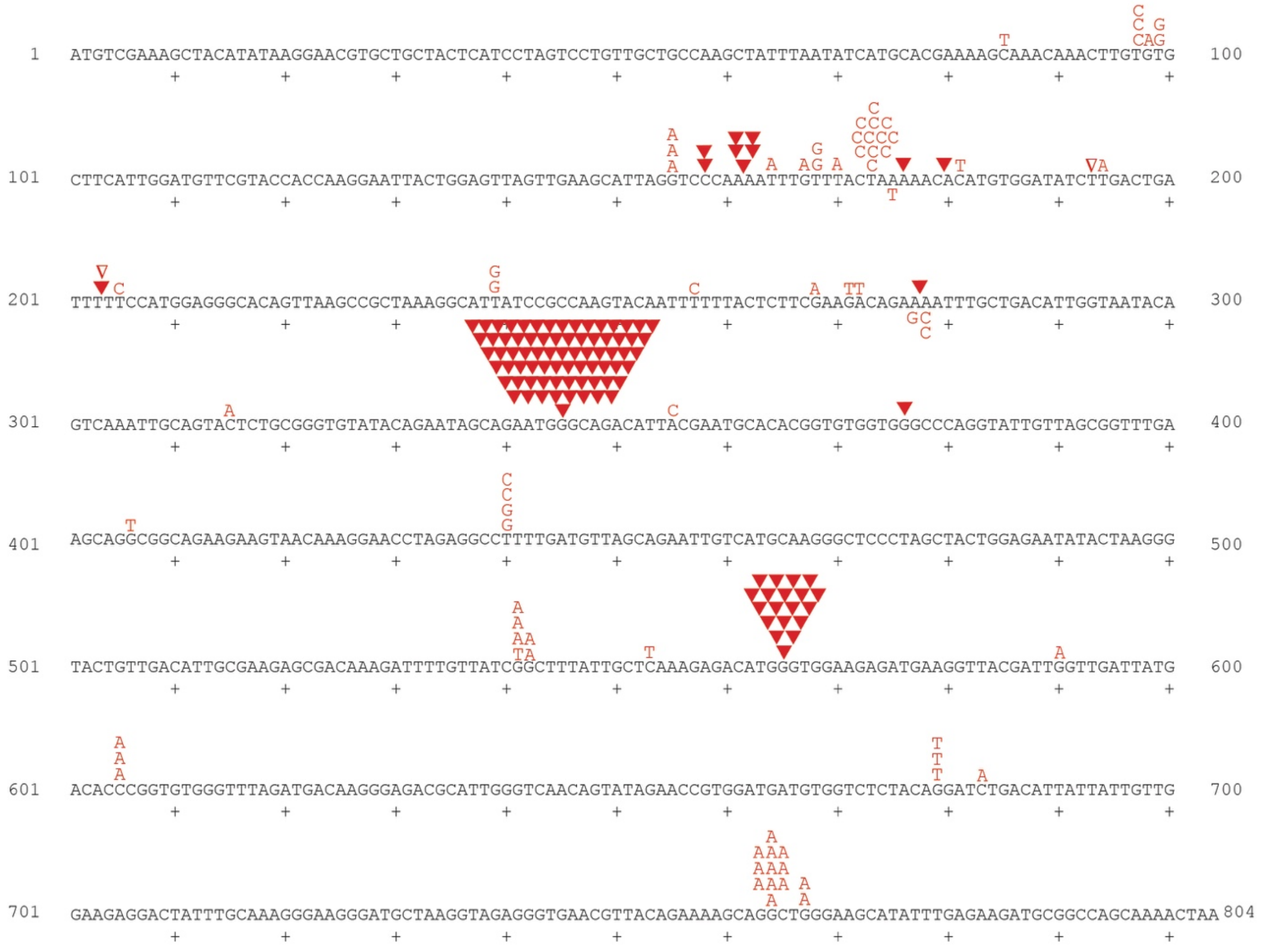

Supplementary Fig. 3. ***URA3*** **mutation spectrum for the** ***cdc9-EE/AA msh6*Δ strain.** As in Supplementary Fig. 2.

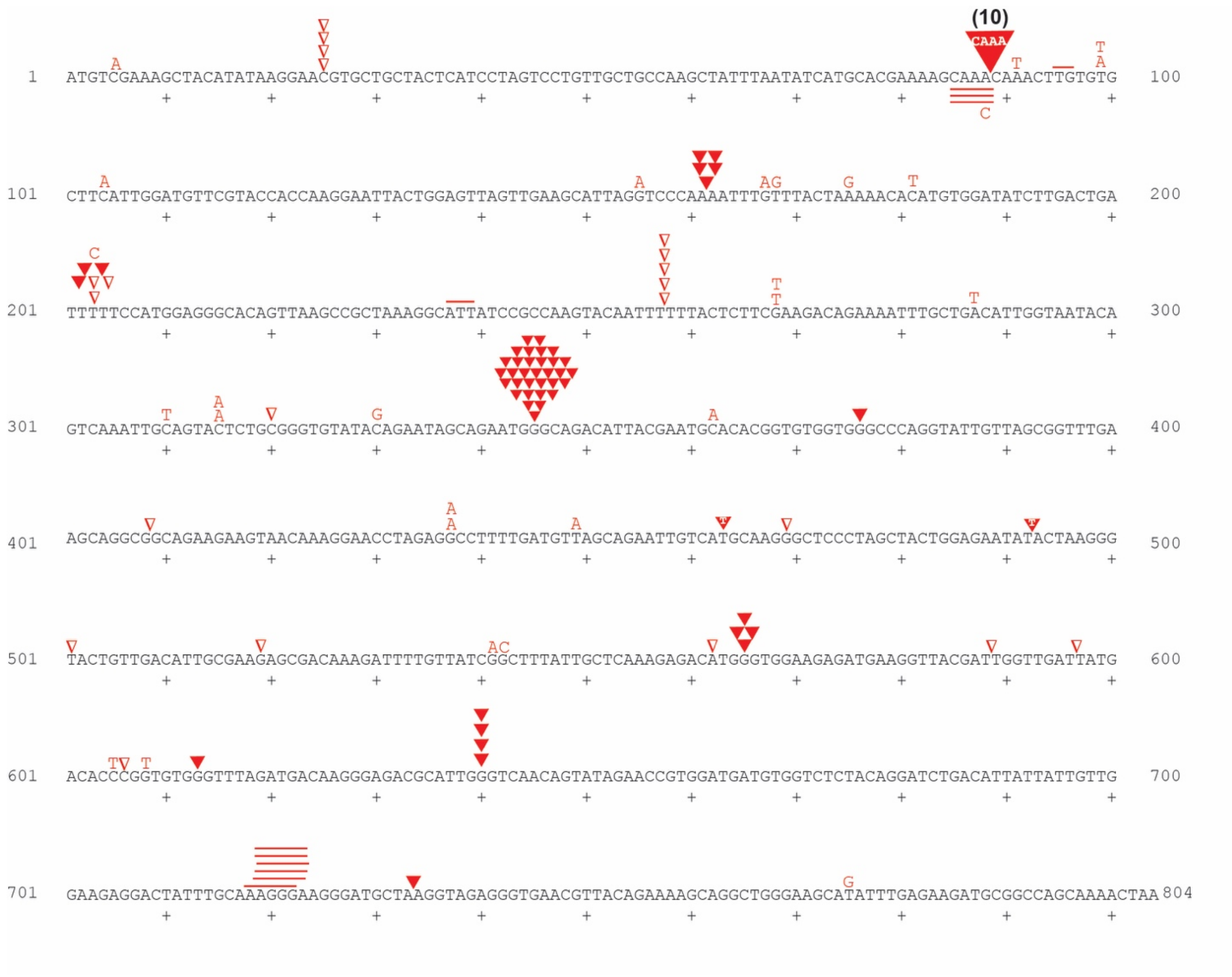

Supplementary Fig. 4. ***URA3*** **mutation spectrum for the** ***cdc9-EE/AA msh3Δ*** **strain.** As in Supplementary Fig. 2. Short lines indicate deletions of between 2 and 5 bases in size.

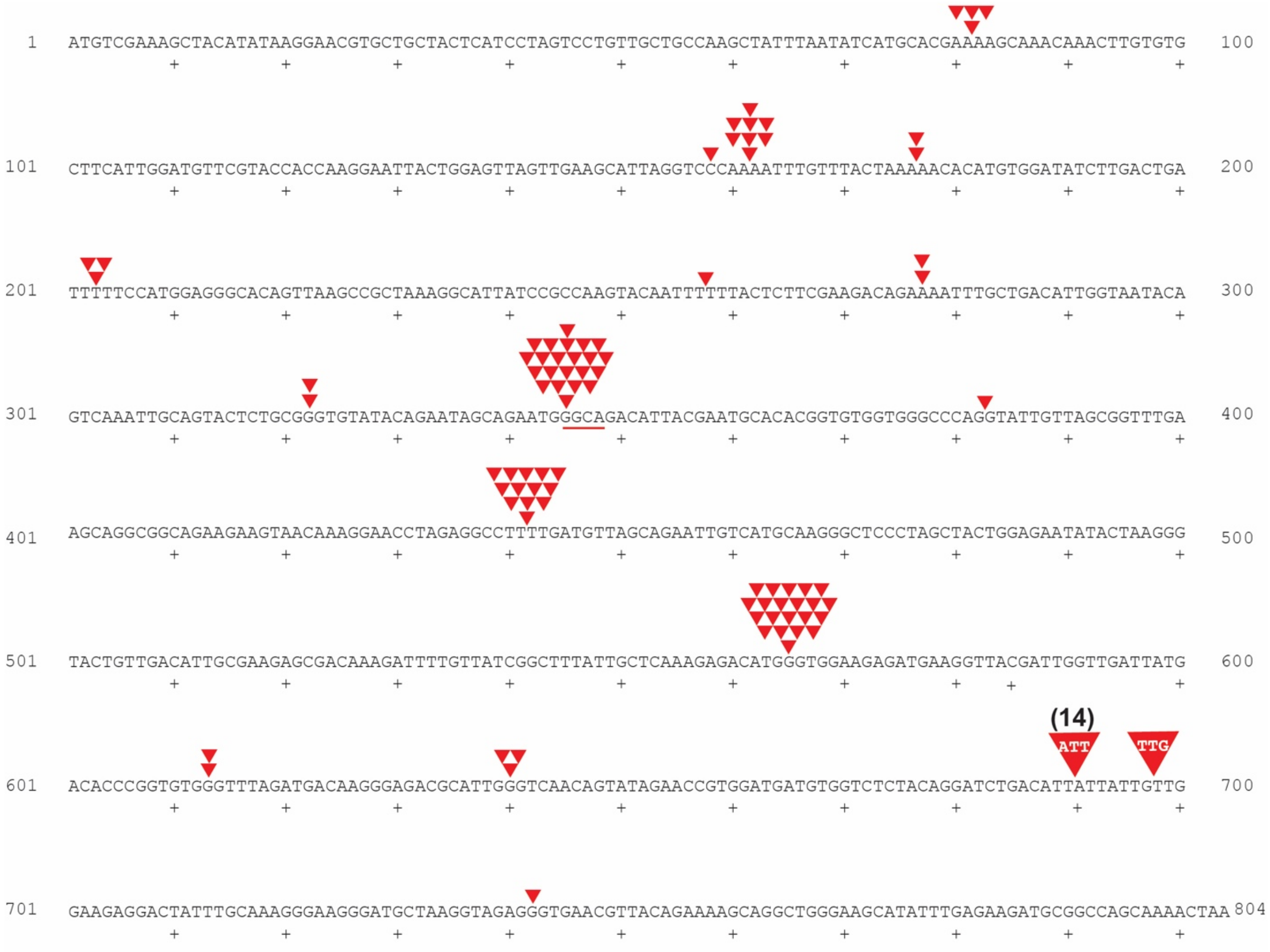

Supplementary Fig. 5. ***URA3*** **mutation spectrum for the** ***cdc9-EE/AA rad27*****Δ strain.** As in Supplementary Fig. 2 and 4.

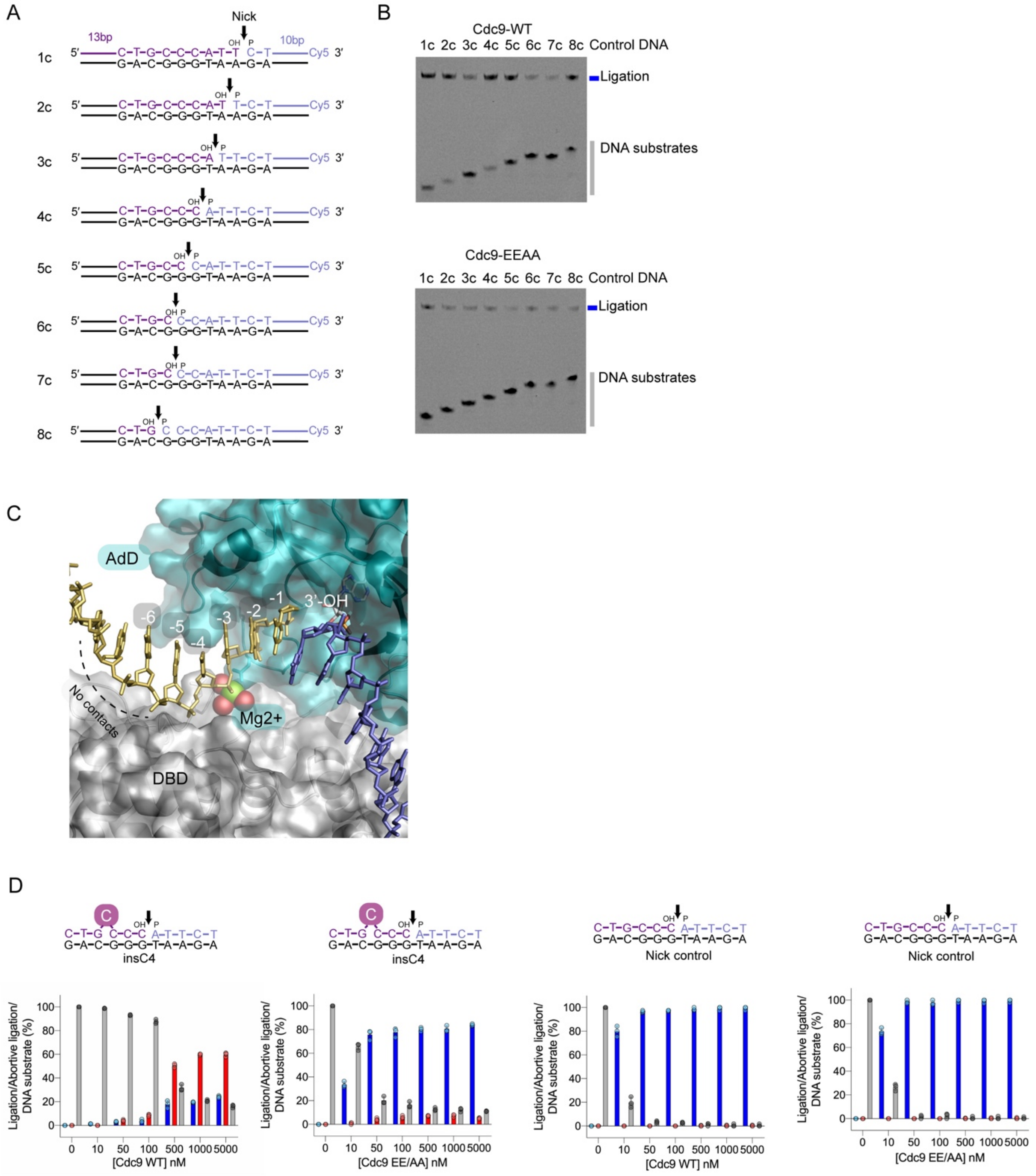

Supplementary Fig. 6. Cdc9 catalytic activity on Control DNA substrates. **a**, Control nick DNA substrates (1c – 8c) are identical to their corresponding insC substrates (Figure 5A) except the absence of a bulged base. **b**, Cdc9 ligation activity on control nick DNA substrates (1c – 8c). Denaturing gel image of ligation reactions in the presence of 10 mM $MgCl_2$, 1 mM ATP, 15 mM NaCl, 50 nM DNA substrate and 2 nM of Cdc9^{WT} (top gel) or Cdc9$^{EE/AA}$ (bottom gel). Source data are provided as a Source Data file. **c**, Surface representation of LIG1^{WT} bound to a nicked

DNA (PDB 6P09). DNA-binding footprint of LIG1 is 5 nucleotides (nt-1 – nt-5) on the 3′OH strand (gold). Nucleotides beyond nt-5 are not contacted by the core domains. **d,** Quantification of total catalytic events on insC4 (left 2 graphs) and control nicked DNA (right 2 graphs) producing ligated product (blue bars) and abortive DNA adenylation (red bars) by $Cdc9^{WT}$ or $Cdc9^{EE/AA}$ analyzed with ImageQuant TL software (GE). Mean ± s.d. (n=3 replicates) is displayed for 20 min ligation reactions. This quantification is the same as in Fig. 5e, but the data are displayed as individual bar graphs for quantification of ligation states, with individual data points shown as individual circles.

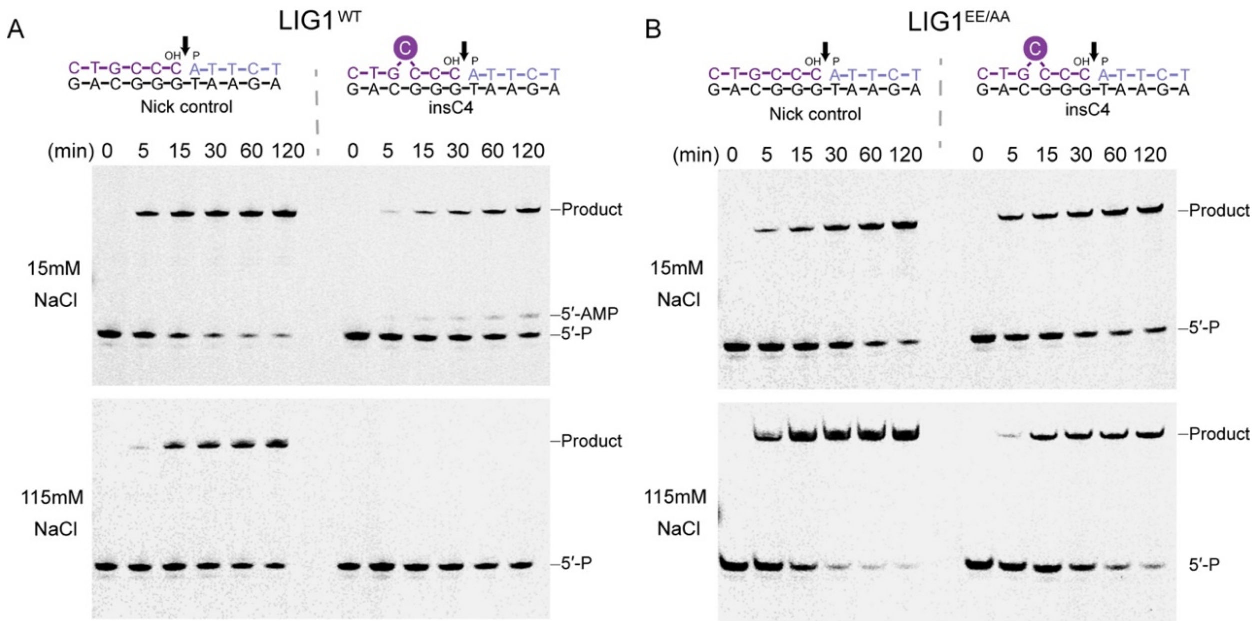

Supplementary Fig. 7. Suppression of hLIG ligation fidelity on insC substrates. **a**, LIG1^{WT} ligation activity on insC4 (right gels) and control nicked DNA substrate c4 (left gels) under low-salt (15 mM, top gels) or high-salt (115 mM, bottom gels) condition. DNA ligation reactions containing 10 mM $MgCl_2$, 1 mM ATP, 15 or 115 mM NaCl, 50 nM DNA substrate, and 2nM LIG1 protein were monitored at the indicated time points. **b**, Low-fidelity mutant LIG1$^{EE/AA}$ retains robust ligation activity on the insC4 substrate despite high stringency reaction conditions. Each of these experiments was repeated independently 3 times with similar results. Source data for panels are provided as a Source Data file.

Supplementary Table 1. Haploid *S. cerevisiae* strains

Strain	Name(s)	Relevant Genotype	Source
wt	SNM8	*agp1::URA3-OR1*	[1]
cdc9-wt	YJW1103,4,9,10	*cdc9-5FLAG:hphMX6*	This study
cdc9-EE/AA	YJW1190,91,92,94	*cdc9-E206A-E443A-5FLAG:hphMX6*	This study
msh2Δ	YJW1506,7,14,15	*msh2::LEU2*	This study
cdc9-EE/AA msh2Δ	YJW1500,04,17,18	*cdc9-E206A-E443A-5FLAG:hphMX6 msh2::LEU2*	This study
msh6Δ	YJW1335,6,41,42	*msh6::LEU2*	This study
cdc9-EE/AA msh6Δ	YJW1331,32,37,38	*cdc9-E206A-E443A-5FLAG:hphMX6 msh6::LEU2*	This study
msh3Δ	YJW1565-69	*msh3::LEU2*	This study
cdc9-EE/AA msh3Δ	YJW1553,57,59,62,64	*cdc9-E206A-E443A-5FLAG:hphMX6 msh3::LEU2*	This study
rad27Δ	JR161-165	*rad27::LEU2*	This study
cdc9-EE/AA rad27Δ	MEA1006,18,80,81	*cdc9-E206A-E443A-5FLAG:hphMX6 rad27::LEU2*	This study

Supplementary Table 2. Mutation events in the *cdc9-EE/AA* strain involving multiple bases

Location	From	To	Type
***URA3* OR1**			
**245-263**	CCAAGTACAATTTTTTACT	CCAAGCCCTTTTTTTACT	Complex
**273-284**	CAGAAAATTTGC	CAGAATATTTTGC	Complex
**454-471**	GAATTGTCATGCAAAGGGC	GAATTGTCATGCAAGGGC	Complex
**277-302**	AATTTGCTGACATTGGTAATACAGT	-	Deletion
**330-337**	CAGAATAG	-	Deletion
**309-336**	-	GCAGTACTCTGCGGGTGTATACAGAATA	Duplication

Supplementary Table 3. Total and specific mutation counts for the *cdc9-EE/AA* mutant strain ± *MMR or RAD27*

Strain	BPS	-1 frameshifts	+1 frameshifts	Total
wt	119	11	0	191
cdc9-EE/AA	41	7	128	189
cdc9-EE/AA msh2Δ	11	30	177	236
msh2Δ	55	100	10	180
Cdc9-EE/AA msh6Δ	75	2	104	186
msh6Δ	292	3	3	691
Cdc9-EE/AA msh3Δ	28	21	53	203
msh3Δ	66	35	1	168
Cdc9-EE/AA rad27Δ	0	0	86	141
rad27Δ	1	3	1	95

The number of events for each mutation category and total mutation counts were used to calculate the specific mutation rates in Fig. 1f. The specific mutation rate of single-base pair substitutions (BPS), -1 frameshifts and +1 frameshifts in each of the indicated strains were calculated as the proportion of each type of event among the total 5-FOA^R mutants sequenced, multiplied by the overall mutation rate (Fig. 1e). The sequencing data for *wt* and *msh2*Δ is from [1] and the sequencing data for *msh6*Δ is from [2].

Supplementary Table 4. Mutation Specificity in the *MSH6* or *MSH3*-deficient strains +/- *cdc9-EE/AA*

Strain	BPS	-1 frameshifts	+1 frameshifts
Cdc9-EE/AA msh6Δ	40	1.1	56
msh6Δ	14	0.14	0.14
Cdc9-EE/AA msh3Δ	2.1	1.6	3.9
msh3Δ	1.7	0.88	0.025

The specific mutation rates ($x10^{-8}$) of single-base pair substitutions (BPS), -1 frameshifts and +1 frameshifts in each of the indicated strains were calculated as the proportion of each type of event among the total mutants sequenced (Supplementary Table 3), multiplied by the overall mutation rate (Fig. 1e).

**Supplementary Table 5. Duplications observed in *cdc9-EE/AA rad27*Δ cells**	*URA3* location	# events observed
gacagaAAATTTGCTGACATTGGTAATACAGTCaaattg	277- 303	4
aagagcGACAAAGATTTGTTATCGGCTTTATTGCTCAAAGAgacatg	523-558	3
tttgctGACATTGGTAATACAGTCAAATTGCAgtactc	286-311	2
gttacgATTGGTTGATTATGACACCCGGTGTGGGTTTAGATGACAAGGGAGACGC attggg	587-635	2
gttggaAGAGGACTATTTGCAAAGGGAAGGGATGCTAAGGTagaggg	703-737	2
ggctggGAAGCATATTTGAgaagat	769-781	2
acagaaAAGCAGGCTGGGaagcat	758-769	1
agagggTGAACGTTACAGAAAAGCAGGCtgggaa	744-765	1
aagggaAGGGATGCTAAGGTAGagggtg	724-739	1
aacagtATAGAACCGTGGATGATGTGGTCTCTACAGGatctga	650-680	1
gcattgGGTCAACAGTATAGAACCGTGGATGATGTggtctc	640-668	1
ttgattATGACACCCGGTGTGGGTTTAGatgaca	598-619	1
ttacgATTGGTTGATTATGACACCCGGTGTGGGTTTAGATGACAAGGGAGACGCATTGGG TCAACAGTATAGAACCGTGGATGATGTGGTCTCTA CAGGATCTGACATTATTattgtt	587-693	1
aagagaTGAAGGTTACGATTGGTTGATTATGACACCCGGTGTGGGTTTAGATGACAAGGG AGACGCATTGGGTCAACAGTATAGAACCGTGGATGATGtggtct	576-667	1
ggaagaGATGAAGGTTACGATTGGTTgattat	574-593	1
tggaagAGATGAAGGTTACGATTGGTTGATTATGACACCCGGTGTGGGTTTagatga	573-617	1
attgcgAAGAGCGACAAAGATTTGTTATCGGCTTTATTGCTCAaagaga	517-554	1
cattgcGAAGAGCGACAAAGATTTGTTATCGGCTTTATTGCTCAAAGAGACATGGGTGga agag	516-568	1
taagggTACTGTTGACATTGCGAAGAGCGACAAAGATTTGTTATCGGCTTTATTGCTCAA AGAGACATGGGTGGAAGAGATGAAGGTtacgat	501-582	1
aggtatTGTTAGCGGTTTGAAGCAGGCGGCAGAAGAAGTAACAAAGGAACCTAGAGGCC TTTTGAtgttag	387-445	1
tggtggGCCCAGGTATTGTTAgcggtt	377-391	1
gcagaaTGGGCAGACATTACGAATGCACACGGTGTGGtgggcc	343-373	1
tgcgggTGTATACAGAATAGCAGAATGGGCAGACATTACGAAtgcaca	324-359	1
tggtaaTACAGTCAAATTGCAGtactct	297-312	1
tgacatTGGTAATACAGTCAAATTGCAGTACTC tgcggg	291-317	1
tttgctGACATTGGTAATACAGTCAAATTGCAGTACTCTGCGGGTGTATACAGAATAGCAG AATGGGCAgacatt	286-348	1
attttTACTCTTCGAAGACAGAAAATTTGCTGACATTGGTAATACAGTCAAATTGCAG tactct	260-312	1
cacgaaAAGCAAACAAACTTGTGTGCTTCATTGGATGTTCGTACCACCAAGGaattac	82-127	1
ggaacgTGCTGCTACTCATCCTAGTCCTGTtgctgc	27-50	1

Independent *cdc9-EE/AA rad27*Δ 5-FOA^{R} revertants were isolated from and sequenced (n = 141), mutations up to 5 bp in size are displayed in Figure S5. Large duplications are shown in uppercase black letters in the first column. Lowercase red letters indicate flanking sequences 5' and 3' of each duplication. Red underlined bases indicate the terminal sequence associated with the duplication. Nucleotide sequence in the *URA3* reporter and number of observed events are listed in columns 2 and 3, respectively. Also observed, a complex mutation TACAGAAAAGCA (751- 762) to TACAAAAACA.

Supplementary Table 6. Breakdown of mutation classes in the *cdc9-EE/AA, cdc9-EE/AA rad27*Δ and *rad27*Δ strains

	Total mutations	BPS	-1 bp frameshifts	+1 bp frameshifts	+2-5 bp frameshifts	Large Duplications	Large deletions	Other
cdc9-EE/AA	189	41	7	128	1	1	2	3
***cdc9-EE/AA rad27*Δ**	141	0	0	86	15	38	1	1
***rad27*Δ**	95	1	3	1	12	68	3	2

The *URA3* reporter from a collection of independent 5-FOA^{R} revertants was sequenced for the *cdc9-EE/AA, cdc9-EE/AA rad27*Δ and *rad27*Δ strains. Shown are the observed counts for each of the specific mutation categories.

Supplementary Table 7. Duplications observed in the *rad27*Δ strain	*URA3* location	# events observed
aagagcGACAAAGATTTTGTTATCGGCTTTATTGCTCAAAGAgacatg	523-558	10
caaattGCAGTACTCTGCGGGTGTATACAGAATAgcagaa	309-336	7
aagggaAGGGATGCTAAGGTAGagggtg	724-739	4
gttggaAGAGGACTATTTGCAAAGGGAAGGGATGCTAAGGTagaggg	703-737	4
ttgattATGACACCCGGTGTGGGTTTAGatgaca	598-619	3
tgaagcAGGCGGCAGAAGAAGTaacaaa	404-419	3
tggtaaTACAGTCAAATTGCAGtactct	297-312	3
ttgctgCCAAGCTATTTAATATCATGCACGAAAAGCAAACAAACTTGTGTGCTTCA TTGGATGTTCGTACCAccaagg	56-121	3
agagggTGAACGTTACAGAAAAGCAGGCtgggaa	744-765	2
gcattgGGTCAACAGTATAGAACCGTGGATGATGTggtctc	640-668	2
gcaggcGGCAGAAGAAGTAACAAAGGAACCTAGAggcctt	408-435	2
tgcacaCGGTGTGGTGGGCCCAGGTATTGTTAGCGGTTTGAAGCAGGCGGCGGAA GAAGTAACAAAGGAACCTAGAGGCCTTTTGATGTTAGCAGAATTGTCATGCAA GGGCTCCCTAGCTACTGGAGAATATACTAAGGGTACTGTTGACATTGCGAAGA GCGACAAAGATTTTGTTATCGGCTTTATTGCTCAAAGAGACATGGGTGGAAGA GATGAAGGTTACGATTGGTTGATTATGACACCcggtgt	366-605	2
gaaaatTTGCTGACATTGGTAATACAGTCAAAttgcag	281-306	2
gacagaAAATTTGCTGACATTGGTAATACAGTCaaattg	277- 303	2
gttaagCCGCTAAAGGCATTATccgcca	226-241	2
ggctggGAAGCATATTTGAgaagat	769-781	1
tgcaaaGGGAAGGGATGCTAAGGTAGAGGGTGAACGTTACAGAAAAGCAGGCTgg gaag	720-766	1
gatgatGTGGTCTCTACAGGATCTGACATTATTATTGTTGGAAGAGgactat	667-706	1
tgattaTGACACCCGGTGTGGGTTTAGATGACAAGGGAGACGCATTGGGTCAACA GTATAGAACCGTGGATGATGTGGTCTCTACAGGATCtgacat	599-683	1
cattgcGAAGAGCGACAAAGATTTTGTTATCGGTTTATTGCTCAAAGAGACATGG GTGgaagag	516-568	1
cctagcTACTGGAGAATATACTAAGGGtactgt	480-500	1
tgaagcAGGCGGCAGAAGAagtaac	404-416	1
cagaatGGGCAGACATTACGAATGCACACGGTGTGGTGGGCCCAGGTATTGTTAG CGGTTTGAAGCAGGCGGCAGAAGAAGTAACAAAGGAACCTAGAGGCCTTTTG ATGTTAGCAGAATTGTCATGCAAgggctc	344-467	1
aggtatTGTTAGCGGTTTGAAGCAGGCGGCAGAAGAAGTAACAAAGGAACCTAGA GGCCTTTTGAtgttag	387-445	1
atagcaGAATGGGCAGACATTACgaatgc	340-356	1
tgtataCAGAATAGCAGAATGGGCAGACATTACGAATGCACAGGTGTGGTGGGCC CAGGTATTGTTAGCGGTTTGAAGCAGGCGGCAGAAGAAGTAACAAAGGAACC TAGAGGCCTTTTGATGTTAGcagaat	330-451	1
gggtgtATACAGAATAGCAGAATGGGCAGACATTACGAATGCacacgg	327-362	1
tgcagtACTCTGCGGGTGTATACAGAATAGCAGAatgggc	314-341	1
tttgctGACATTGGTAATACAGTCAAATTGCAgtactc	286-311	1
tcgaagACAGAAAATTTGCTGACATTGGTAATacagtc	272- 297	1

agttagTTGAAGCATTAGGTCCCAAAATTTGTTTACTAAAAACACATGTGGATATC ttgact	143-192	1
ggaacgTGCTGCTACTCATCCTAGTCCTGTtgctgc	27-50	1

Independent *rad27*Δ 5-FOA^R revertants were isolated and sequenced (n = 95). Large duplications are shown in uppercase black letters in the first column. Lowercase red letters indicate flanking sequences 5' and 3' of each duplication. Red underlined bases indicate the terminal sequence associated with the duplication. Nucleotide sequence in the *URA3* reporter and number of observed events are listed in columns 2 and 3, respectively. Also observed were the following: a complex mutation ATGTCGAA (1-8) to ATGCACGA, a complex mutation at position 105 involving non-URA3 sequence until position 138 and three large deletions that spanned positions 37-57, 464-540 and 448-622.

Supplementary Table 8. Hotspot analysis of +1 G/C insertions in the low-fidelity *cdc9-EE/AA* mutant strain ± *MSH2*

Strain	Mutation counts of +G/C insertions at *URA3* positions			
	**157**	**344**	**564**	**612**
wt	0	0	0	0
cdc9-EE/AA	4	87	9	7
***cdc9-EE/AA msh2*Δ**	29	68	16	26
***msh2*Δ**	0	0	0	0
***cdc9-EE/AA rad27*Δ**	1	22	21	2
***rad27*Δ**	0	0	0	0

Mutation counts of +1 C/G insertions at each of the indicated hotspots in *URA3*.

Supplementary Table 9. Data collection and refinement statistics

	$LIG1^{EE/AA}$-bulged DNA	$LIG1^{EE/AA}$-unbulged DNA
PDB entry ID	7KR3	7KR4
**Data collection**		
Wavelength	1.000	1.000
Resolution range (Å)	50-2.78 (2.85-2.78)	50-2.20 (2.24-2.20)
Space group	$P2_12_12_1$	$P2_12_12_1$
Cell dimensions:		
a, *b*, *c* (Å)	73.68, 101.67, 115.50	72.37, 96.56, 115.00
α, β, γ (°)	90, 90, 90	90, 90, 90
Unique reflections	21959 (1097)[1]	42631 (2078)[2]
Completeness (%)	97.5 (98.1)	99.7 (99.6)
Redundancy	6.0 (5.5)	6.3 (6.7)
R_{sym}	0.066 (0.422)	0.113 (0.458)
Mean $I/\sigma (I)$	21.5 (1.6)	15.7 (2.0)
**Refinement**		
Resolution (Å)	36.84-2.78	37.92-2.20
Reflections: work/free	21866 / 1998	41440 / 1948
R_{work} / R_{free}	0.193 / 0.239	0.162 / 0.215
Non-H atoms:		
Protein/DNA	5689	5696
AMP	23	23
Solvent	17	321
Average B-factors (Å^2):		
Protein/DNA	87.94	41.85
AMP	67.11	33.02
Solvent	73.09	46.77
R.M.S.D		
Bond lengths (Å)	0.002	0.012
Bond angles (°)	0.51	1.16

Each data set was collected from a single crystal.
[1]Values in parentheses are for highest-resolution shell (2.85-2.78 for $LIG1^{EE/AA}$-bulged DNA).

[2]Values in parentheses are for highest-resolution shell (2.24-2.20) $LIG1^{EE/AA}$-unbulged DNA).

Supplementary Table 10. Insertion and Control DNA oligonucleotides

Insertion and Control DNA oligonucleotides			
**Substrate Name**	Bottom template strand	Top 5′-3′ strand	Top 5′-3′ Cy5 labelled strand
**Insertion DNA 1**	5′-ACAGAATAGCAGAATGGGCAGACATTACGAATGC-3′	5′-GCATTCGTAATGTCTGCCCCATT-3′	5′-phosphate-CTGCTATTCTGT-Cy5-3′
**Insertion DNA 2**	5′-ACAGAATAGCAGAATGGGCAGACATTACGAATGC-3′	5′-GCATTCGTAATGTCTGCCCCAT-3′	5′-phosphate-TCTGCTATTCTGT-Cy5-3′
**Insertion DNA 3**	5′-ACAGAATAGCAGAATGGGCAGACATTACGAATGC-3′	5′-GCATTCGTAATGTCTGCCCCA-3′	5′-phosphate-TTCTGCTATTCTGT-Cy5-3′
**Insertion DNA 4**	5′-ACAGAATAGCAGAATGGGCAGACATTACGAATGC-3′	5′-GCATTCGTAATGTCTGCCCC-3′	5′-phosphate-ATTCTGCTATTCTGT-Cy5-3′
**Insertion DNA 5**	5′-ACAGAATAGCAGAATGGGCAGACATTACGAATGC-3′	5′-GCATTCGTAATGTCTGCCC-3′	5′-phosphate-CATTCTGCTATTCTGT-Cy5-3′
**Insertion DNA 6**	5′-ACAGAATAGCAGAATGGGCAGACATTACGAATGC-3′	5′-GCATTCGTAATGTCTGCC-3′	5′-phosphate-CCATTCTGCTATTCTGT-Cy5-3′
**Insertion DNA 7**	5′-ACAGAATAGCAGAATGGGCAGACATTACGAATGC-3′	5′-GCATTCGTAATGTCTGC-3′	5′-phosphate-CCCATTCTGCTATTCTGT-Cy5-3′
**Insertion DNA 8**	5′-ACAGAATAGCAGAATGGGCAGACATTACGAATGC-3′	5′-GCATTCGTAATGTCTG-3′	5′-phosphate-CCCCATTCTGCTATTCTGT-Cy5-3′
**Control DNA 1c**	5′-ACAGAATAGCAGAATGGGCAGACATTACGAATGC-3′	5′-GCATTCGTAATGTCTGCCCATT-3′	5′-phosphate-CTGCTATTCTGT-Cy5-3′
**Control DNA 2c**	5′-ACAGAATAGCAGAATGGGCAGACATTACGAATGC-3′	5′-GCATTCGTAATGTCTGCCCAT-3′	5′-phosphate-TCTGCTATTCTGT-Cy5-3′
**Control DNA 3c**	5′-ACAGAATAGCAGAATGGGCAGACATTACGAATGC-3′	5′-GCATTCGTAATGTCTGCCCA-3′	5′-phosphate-TTCTGCTATTCTGT-Cy5-3′
**Control DNA 4c**	5′-ACAGAATAGCAGAATGGGCAGACATTACGAATGC-3′	5′-GCATTCGTAATGTCTGCCC-3′	5′-phosphate-ATTCTGCTATTCTGT-Cy5-3′
**Control DNA 5c**	5′-ACAGAATAGCAGAATGGGCAGACATTACGAATGC-3′	5′-GCATTCGTAATGTCTGCC-3′	5′-phosphate-CATTCTGCTATTCTGT-Cy5-3′
**Control DNA 6c**	5′-ACAGAATAGCAGAATGGGCAGACATTACGAATGC-3′	5′-GCATTCGTAATGTCTGC-3′	5′-phosphate-CCATTCTGCTATTCTGT-Cy5-3′
**Control DNA 7c**	5′-ACAGAATAGCAGAATGGGCAGACATTACGAATGC-3′	5′-GCATTCGTAATGTCTGC-3′	5′-phosphate-CCATTCTGCTATTCTGT-Cy5-3′
**Control DNA 8c**	5′-ACAGAATAGCAGAATGGGCAGACATTACGAATGC-3′	5′-GCATTCGTAATGTCTG-3′	5′-phosphate-CCCATTCTGCTATTCTGT-Cy5-3′

Supplementary Table 11. Crystallization oligonucleotides

Crystallization oligonucleotides				
**Oligo number**	Oligo Name	5′ modification	Sequence	3′ modification
**1**	18merDown	none	5’-GCAGAATGGGCAGACATT-3’	none
**2**	12mer3’ddCUp(C-Insertion)	none	5’-AATGTCTGCCCC-3’	ddC
**3**	11mer3’ddCUp	none	5’-AATGTCTGCCC-3’	ddC
**4**	7mer5’PDown	phosphate	5’-ATTCTGC-3’	none

Supplementary References

1. Nick McElhinny, S.A., Kissling, G.E. & Kunkel, T.A. Differential correction of lagging-strand replication errors made by DNA polymerases {alpha} and {delta}. *Proc Natl Acad Sci U S A* **107**, 21070-5 (2010).
2. St Charles, J.A., Liberti, S.E., Williams, J.S., Lujan, S.A. & Kunkel, T.A. Quantifying the contributions of base selectivity, proofreading and mismatch repair to nuclear DNA replication in Saccharomyces cerevisiae. *DNA Repair (Amst)* **31**, 41-51 (2015).
