# Supplementary material for: High-fidelity DNA ligation enforces accurate Okazaki fragment maturation during DNA replication
Source: Nat Commun. 2021 Jan 20;12:482. doi: 10.1038/s41467-020-20800-1 (PMC7817679; doi:10.1038/s41467-020-20800-1)
Supplement: Supplementary file 3 — Description of Additional Supplementary Files [file 41467_2020_20800_MOESM3_ESM.pdf]

Description of Additional Supplementary Files

File Name: Supplementary Movie 1
Description: A structural morph between the un-bulged and bulged DNA bound states of mutant LIG1$^{EE/AA}$ DNA complexes is displayed. The bulged extrahelical nucleotide in the insC complex flips into a pocket created by alanine mutations (sticks) in the AdD (turquoise) and DBD (grey) domains.
